# The association between hobby engagement and depressive symptoms among Chinese middle-aged and older adults: evidence from the China health and retirement longitudinal study

**DOI:** 10.3389/fpubh.2024.1450358

**Published:** 2024-09-27

**Authors:** Qiong Lu, Peijing Hu, Cheng Lian, Xinglin Chen

**Affiliations:** ^1^Quyi Research Institute, Chinese National Academy of Arts, Beijing, China; ^2^Academic Department, Chinese National Academy of Folk Art, Beijing, China; ^3^Department of Cardiology, The Second Affiliated Hospital of Xi'an Medical University, Xi'an, China; ^4^Department of Cardiology, Xi’an, The Affiliated Hospital of Northwest University, Xi’an, Shaanxi, China; ^5^Department of Epidemiology and Biostatistics, Empower U, X&Y Solutions Inc., Boston, MA, United States

**Keywords:** hobby, depression, logistic regression, longitudinal study, adults

## Abstract

**Background:**

It has been demonstrated that receptive cultural engagement, such as visits to theaters and museums, can reduce depression in older adults. However, the association between hobby engagement and lower rates of depressive symptoms (DS) remains unclear. The objective of this study was to investigate the potential relationship between hobby engagement and depressive symptoms.

**Methods:**

The data for this prospective cross-sectional study were collected from participants in the China Longitudinal Study of Health and Retirement (CHARLS) wave 2020. To assess DS, a score of 10 or more on the Center for Epidemiological Studies Depression Scale was used to indicate depression. Hobby engagement was gaged by trained staff through the administration of a battery of standardized questions. Logistic regression and inverse probability of treatment weighting (IPTW) using the propensity score analyses were employed to investigate the relationship between hobby engagement and DS.

**Results:**

A total of 16,057 participants were included, with a mean age of 62.4 ± 9.2 years. Of these, 7,699 were male and 8,358 were female. The proportion of individuals exhibiting depressive symptoms was 31.57% (1,286 out of 4,073) among those with hobby engagement, while it was 39.67% (4,754 out of 11,984) among those without hobby engagement. After adjusting for covariates, the odds ratio was 0.89 (95% CI: 0.82–0.97, *p* = 0.0109). Propensity-score analyses also supported these findings, with the odds ratio being 0.91 (95% CI: 0.84–0.99, *p* = 0.0204). The finding was consistent with multiple sensitivity analyses.

**Conclusion:**

Our study found hobby engagement was associated with a reduced risk of depressive symptoms in Chinese middle-aged and older adults. Our findings need to be confirmed in future studies.

## Introduction

Population aging is a growing global concern. As the population ages, the number of lonely adults aged 50 years and older worldwide is projected to increase from 104.9 million in 1990 to 333.5 million in 2050 (1). A study has indicated that by 2030, the number of older adult people in China aged 60 and above will reach 360 million, representing 16% of the total population (2).

Among middle-aged and older adults, depression is a prevalent mental health issue (3). A number of studies have indicated that the prevalence of depression is higher in the older adult population. This may be related to a number of factors, including the life stressors that they face, social isolation, and concerns about future care needs (3, 4). It is crucial to acknowledge that although depression is more prevalent in older individuals, this does not imply that all older adults experience depression. Indeed, the majority of older adults continue to exhibit a positive outlook on life and experience lower levels of emotional distress (5). Consequently, the reducation or prevention of depression in the older adult is a significant objective (6). There is a growing global focus on depression and coping strategies in older adults, with a particular emphasis on psychosocial activities (7). In this context, numerous countries, including the United Kingdom, Japan, and the United States, have been advocating for the promotion of hobbies and leisure activities as part of policies and recommendations designed to support and enhance mental health. These initiatives have placed a particular emphasis on increasing engagement among older individuals.

A recent study examined the longitudinal association between engagement in hobbies and multidimensional aspects of mental health in 16 countries. Hobbies defined as activities that people engage in during their leisure time for pleasure, such as the arts, crafts, reading, playing games, sports, gardening, volunteering and participating in societies/clubs (8). A study of individuals over the age of 60 revealed considerable variation in the level of participation in hobbies across countries. A meta-analysis of the findings indicated that engaging in a hobby was associated with a reducation in depressive symptoms (8).

The relationship between hobbies and depression is influenced by a number of factors. For instance, a study revealed that the co-occurrence of depression and anxiety was 29.7% in the older population, with women exhibiting a higher prevalence of these disorders than men (9). Furthermore, research has demonstrated that depression among the older adult is not solely attributable to physical health conditions, but also to psychosocial factors such as the death of a spouse, retirement, social isolation, and reduced income (10). A substantial body of evidence from empirical studies indicates that physical activity and nutrition have a pronounced impact on cognitive health and quality of life in older adults. Zhang et al. highlighted the efficacy of appropriate physical activity and nutritional intake in enhancing cognitive ability and quality of life in the older adult, underscoring the significance of lifestyle intervention in geriatric health management (11). Furthermore, Faraziani et al. delved into the potential of exercise-based health management systems in bolstering cognitive ability and postponing cognitive decline in older adults, emphasizing the necessity and efficacy of tailored exercise interventions (12).

Furthermore, the relationship between hobbies and depression remains a topic of contention. Firstly, research has demonstrated that engagement in specific activities, such as music and art, can result in a reducation in depressive symptoms (13). Nevertheless, the aforementioned positive effect does not imply that all forms of hobbies yield identical positive outcomes. For instance, research has indicated that enthusiastic involvement with music may result in the experience of negative emotions (14). This indicates that not all types of hobbies are conducive to positive mental states.

In conclusion, the relationship between hobbies and depression is complex and the subject of considerable debate, with a number of confounding factors influencing the outcome. Based on the above uncertainty, we conducted a prospective cross-sectional study in 450 Chinese communities based on the China Health and Retirement Longitudinal Study (CHARLS) to examine the association between hobby engagement and depressive symptoms in Chinese middle-aged and older adults.

## Methods

### Study design and population

The data for this study were based on the fourth wave of the China Health and Retirement Longitudinal Study (CHARLS) conducted by Peking University in 2020. The project was a nationwide questionnaire survey conducted in 150 counties among 28 provinces (autonomous regions and municipalities) in China (15), established by the National Development Research Institute of Peking University. Briefly, participants were included in the baseline survey between June 2011 and March 2012 of middle-aged and older adults who were randomly selected using a probability proportional to size sampling strategy. CHARLS carried out four national follow-up surveys in 2013, 2015, 2018, and 2020. The 2020 wave of CHARLS data is the most recent data available. The questionnaires and physical examinations were carried out on 19,395 participants in 2020. The CHARLS has been approved by the Ethics Committee of Peking University Health Science Center. The Biomedical Ethics Review Board of Peking University approved the CHARLS study (IRB00001052-11015), and all participants provided written informed consent (15). Data from this study and related information can be downloaded from the CHARLS project website.^1^

**Figure 1:**
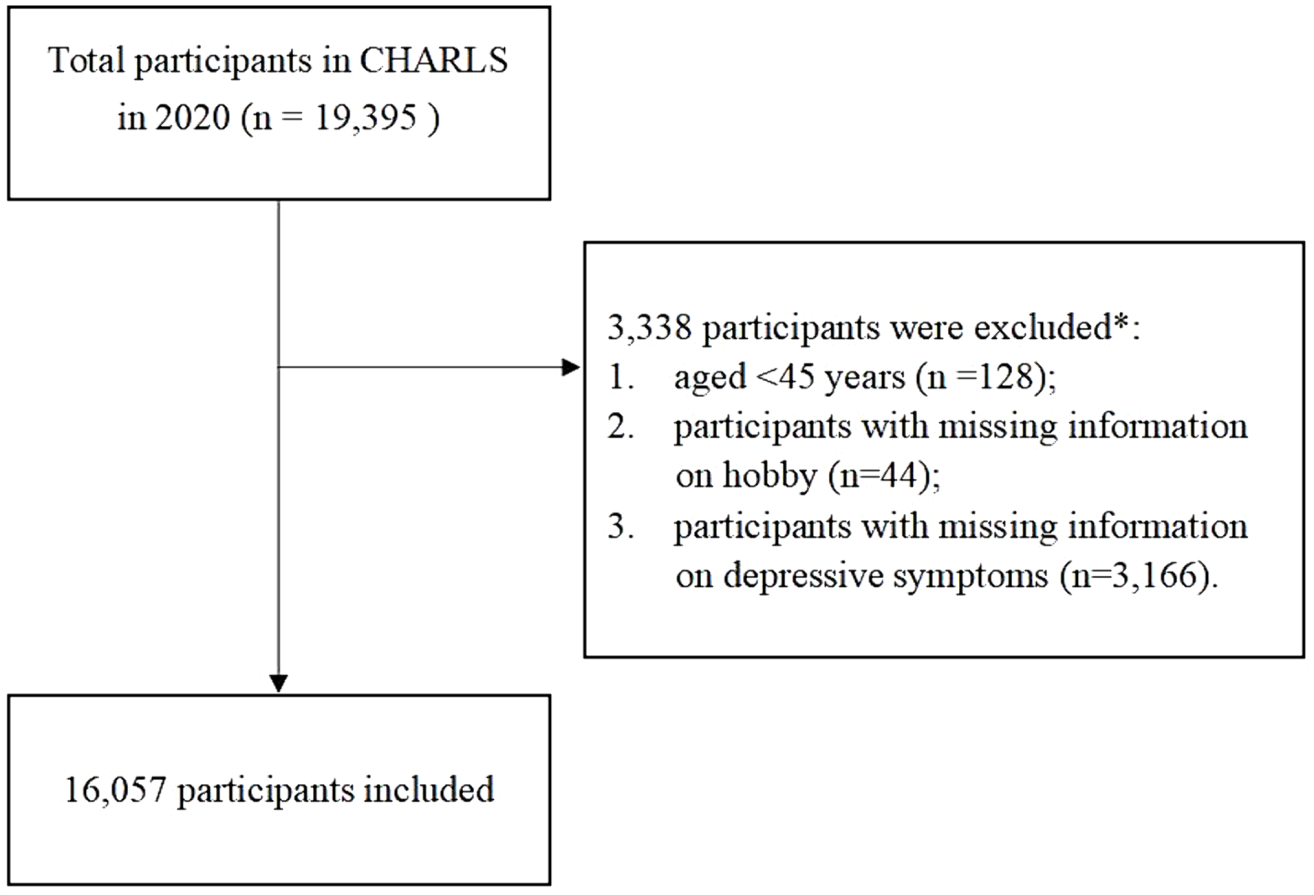
Flowchart of participant selection. *Subjects selected after each step.

For the present study, the following exclusion criteria were applied: (1) aged <45 years old at wave 5 (*n* = 128), (2) participants with missing information on hobby (*n* = 44), (3) participants with missing information on depressive symptoms (*n* = 3,166). A total of 16,057 individuals meeting these criteria were enrolled in the final cohort (Figure 1). The current study follows the Strengthening the Reporting of Observational Studies in Epidemiology (STROBE) reporting guidelines for cohort studies.

### Study variables

#### Assessment of hobby

Hobby is the independent variable of this study as described in another study based on CHARLS (8). As previously reported, trained staff asked participants the following standardized questions: ‘Which of the social activities listed on this card—if any—have you done in the past month?’ 1. Played ma-jong, played chess, played cards or went to a community club. 2. Went to a sport, social or other kind of club. 3. Took part in a community-related organization. 4. Did voluntary or charity work. 5. Attended an educational or training course. 0. None of these [1 = any of the activities; 0 = none]. We created a binary indicator of hobby engagement (yes, no) based on the questionnaire.

#### Assessment of depressive symptoms

The outcome of the study was depressive symptoms. In the CHARLS, depressive symptoms were assessed using the 10-item Center for Epidemiological Studies Depression Scale (CESD-10) (16). The validity of CESD-10 has been thoroughly demonstrated in the Chinese population (Cronbach α = 0.78) (17, 18). Survey respondents were asked about the number of days of relevant experience in the past week. Each item was rated on a four-point Likert scale: 0 (rarely or none of the time; < 1 day), 1 (some of the time; 1–2 days), 2 (much or a moderate amount of the time; 3–4 days), or 3 (most or all of the time; 5–7 days). This study assigned 0–3 to these four options, with a total depressive symptoms score ranging from 0 to 30, with higher scores indicating higher levels of depressive symptoms. The cut-off points of 10 used in CESD-10 have been validated in numerous studies of the older adults in China (19–21). Thus, we used a total score of ≥10 was defined as depressive symptoms.

### Other covariates

Based on the well-designed questionnaire, the CHARLS-trained interviewers collected information on demographic background, health status, and biomarkers. Covariates were selected on the basis of findings from previous studies and clinical expertise (22, 23). The variables included in this study were age, sex, smoking status (never, current and ever), drinking status (never, current and ever), educational attainment (primary and below primary school, middle school, and high school and above), area of residence (urban and rural), and marital status (married and other), which were collected from each participant in a face-to-face interview as previously described (15). Standing height was measured by a standardized stadiometer (Seca™213, Seca Co., Ltd., Hangzhou, China) and the weight was assessed by a validated scale (Omron™ HN-286 scale, Krill Technology Co., Ltd., Yangzhou, China). BMI was calculated as weight (kg) divided by the square of height (m^2^). Chronic diseases included hypertension, diabetes mellitus (DM) or high blood sugar. Hypertension was defined as SBP ≥140 mmHg and/or DBP ≥90 mmHg or being on antihypertensive therapy. DM was defined as FPG ≥7.0 mmol/L or a history of physician-diagnosed DM.

### Statistical analysis

Statistical analyses were conducted using the EmpowerStats (X&Y solutions, Inc. Boston MA)^2^ and R software version 3.6.1.^3^ The two-sided alpha level was set at 0.05. Continuous variables were presented using either the mean ± standard deviation (SD; for normally distributed data) or median interquartile ranges (IQR; for skewed distribution). Categorical data are presented as counts with percentages.

The differences between hobby engagement groups was compared using T test (for normally distributed data), the Kruskal–Wallis H test (for skewed data) for continuous data and chi-squared tests for categorical variables.

Logistic regression model analyses were used. The results are presented as odds ratio (OR) with its 95% confidence intervals (95% CIs). Covariates were selected according to their clinical significance, including demographic characteristics, comorbidities, and general data for the following covariates: age, sex, marital status, education, self-reported health, hypertension, diabetes mellitus, heart disease, stroke, arthritis, dyslipidemia, hepatic disease, sleep duration, residence, and annual income.

To address the potential confounding effect, a propensity score (PS) approach was applied. Optimal full matching was performed using the R Matchit package, with the distance metric defined as the PS calculated from the generalized additive model. To estimate the treatment effect following full matching, the generalized additive model with logit as link function was employed. The way standard errors and confidence intervals were estimated using cluster-robust standard errors with pair membership as the cluster, R vcovCL() in sandwich package was used.

Sensitivity analysis was utilized to test the robustness of our findings. (1) Inverse probability of treatment weighting (IPTW) using the propensity score analyses were employed to investigate the relationship between hobby engagement and DS. Within the IPTW analysis, the predicted probabilities derived from the propensity score model were utilized to calculate the IPTW (24). (2) To examine the robustness of our results, stratified analyses of all covariates were performed. (3) Dummy variables were used to indicate missing covariate values. (4) The E-value was calculated to assess the possibility of unmeasured confounders between green space and stroke risk (25). The E-value quantifies the amount of unmeasured confounding that is required to eliminate the observed association between hobby engagement and DS. (5) Previous studies have shown a significant association between hypertension and depression (26). Participants without hypertension (*n* = 9,819) were analyzed.

## Results

### Characteristics of the participants

A total of 16,057 individuals with a mean age of 62.4 ± 9.2 years were included in the study. Table 1 presents the characteristics and depressive symptoms of participants in the China Health and Retirement Longitudinal Study 2020, stratified by hobby engagement. The table includes key variables such as age, sex, residence, marital status, education, annual income, self-reported health status, and various health conditions. Participants engaged in hobbies had a lower mean age (61.45 years) compared to those not engaged (62.78 years). A higher percentage of participants in the hobby engagement group reported good self-reported health status (27.96%) compared to the non-engagement group (24.35%). Furthermore, the prevalence of depressive symptoms was lower in the hobby engagement group (31.57%) compared to the non-engagement group (39.67%), with both results being statistically significant (*p* < 0.05). Furthermore, the table reveals significant discrepancies in variables such as age, sex, place of residence, marital status, level of education, annual income, self-reported health status, hypertension, diabetes mellitus, heart disease, stroke, arthritis, dyslipidemia, hepatic disease, duration of sleep, and depressive symptoms between the two groups (all *p* < 0.05). These findings indicate that the above variables were associated with depression, and thus were adjusted in the subsequent analysis.

**Table 1:** Characteristics and depressive symptoms according to hobby engagement (China Health and Retirement Longitudinal Study 2020).

Variable	All participants	Hobby engagement		
		No	Yes	*p*-value
N	16,057	11,984	4,073	
Age (years)	62.44 ± 9.19	62.78 ± 9.34	61.45 ± 8.65	<0.001
Age groups				
< 60	6,951 (43.29%)	5,040 (42.06%)	1911 (46.92%)	<0.001
60–70	5,397 (33.61%)	3,995 (33.34%)	1,402 (34.42%)	
> =70	3,709 (23.10%)	2,949 (24.61%)	760 (18.66%)	
Sex (%)				<0.001
Female	8,358 (52.05%)	6,349 (52.98%)	2009 (49.32%)	
Male	7,699 (47.95%)	5,635 (47.02%)	2064 (50.68%)	
Residence (%)				<0.001
Urban	6,456 (40.21%)	4,377 (36.52%)	2079 (51.04%)	
Rural	9,601 (59.79%)	7,607 (63.48%)	1994 (48.96%)	
Marital status (%)				<0.001
No spouse	2,227 (13.87%)	1744 (14.55%)	483 (11.86%)	
Married with spouse	13,830 (86.13%)	10,240 (85.45%)	3,590 (88.14%)	
Education (%)				<0.001
Illiterate	6,340 (39.48%)	5,224 (43.59%)	1,116 (27.40%)	
Primary	3,678 (22.91%)	2,758 (23.01%)	920 (22.59%)	
Middle	3,840 (23.91%)	2,660 (22.20%)	1,180 (28.97%)	
College or higher	2,199 (13.69%)	1,342 (11.20%)	857 (21.04%)	
Annual income (CNY)	30,360 (6690–66,900)	26,507 (5740–61,000)	42,120 (12000–80,000)	<0.001
Annual income groups				<0.001
< 15,000	5,166 (32.17%)	4,155 (34.67%)	1,011 (24.82%)	
> =15,000, <25,000	1,281 (7.98%)	990 (8.26%)	291 (7.14%)	
> =25,000	7,842 (48.84%)	5,473 (45.67%)	2,369 (58.16%)	
Missing	1768 (11.01%)	1,366 (11.40%)	402 (9.87%)	
Self-reported health status (%)				<0.001
Good	4,056 (25.26%)	2,917 (24.35%)	1,139 (27.96%)	
Fair	8,148 (50.75%)	6,024 (50.28%)	2,124 (52.15%)	
Poor	3,850 (23.98%)	3,040 (25.37%)	810 (19.89%)	
Hypertension (%)				0.022
No	9,819 (61.15%)	7,267 (60.64%)	2,552 (62.66%)	
Yes	6,238 (38.85%)	4,717 (39.36%)	1,521 (37.34%)	
Disabetes or hyperglycaemia (%)				0.069
No	13,713 (85.40%)	10,270 (85.70%)	3,443 (84.53%)	
Yes	2,344 (14.60%)	1714 (14.30%)	630 (15.47%)	
Heart disease (%)				0.403
No	12,705 (79.12%)	9,501 (79.28%)	3,204 (78.66%)	
Yes	3,352 (20.88%)	2,483 (20.72%)	869 (21.34%)	
Stroke (%)				0.186
No	15,053 (93.75%)	11,217 (93.60%)	3,836 (94.18%)	
Yes	1,004 (6.25%)	767 (6.40%)	237 (5.82%)	
Arthritis (%)				<0.001
No	9,885 (61.56%)	7,233 (60.36%)	2,652 (65.11%)	
Yes	6,172 (38.44%)	4,751 (39.64%)	1,421 (34.89%)	
dyslipidemia (%)				<0.001
No	11,721 (73.00%)	8,903 (74.29%)	2,818 (69.19%)	
Yes	4,336 (27.00%)	3,081 (25.71%)	1,255 (30.81%)	
Hepatic disease (%)				<0.001
No	14,878 (92.66%)	11,165 (93.17%)	3,713 (91.16%)	
Yes	1,179 (7.34%)	819 (6.83%)	360 (8.84%)	
Sleep duration (h)	6.03 ± 1.89	6.00 ± 1.96	6.10 ± 1.69	0.004
Sleep duration groups				<0.001
< 6	5,740 (35.75%)	4,387 (36.61%)	1,353 (33.22%)	
> =6	10,317 (64.25%)	7,597 (63.39%)	2,720 (66.78%)	
Depressive symptoms CESD-10	8.63 ± 6.45	8.97 ± 6.58	7.63 ± 5.97	<0.001
Depressive symptoms				<0.001
No	10,017 (62.38%)	7,230 (60.33%)	2,787 (68.43%)	
Yes	6,040 (37.62%)	4,754 (39.67%)	1,286 (31.57%)	

Data are expressed as mean ± SD, median (25th-75th percentile) or percentage. Among the 16,057 participants, the number of missing values for the covariates was 1,768 (11.0%) for annual income and 3 (<1.0%) for self-reported health status.

### Factors influencing the risk of depressive symptoms analyzed by logistics regression

Table 2 presents the associations between various variables and depressive symptoms among participants in the China Health and Retirement Longitudinal Study 2020. The table displays the odds ratios (OR) and 95% confidence intervals (CI) for each exposure variable in relation to depressive symptoms. Those who engaged in hobbies were found to have a lower odds of depressive symptoms, with an OR of 0.70 (95% CI: 0.65, 0.76). This demonstrated a statistically significant association between hobbies and a 30% reducation in depressive symptoms. In contrast, age was positively associated with depressive symptoms, with an OR of 1.02 (95% CI: 1.01, 1.02). Furthermore, variables such as sex, marital status, education, self-reported health status, hypertension, diabetes mellitus, heart disease, stroke, arthritis, dyslipidemia, hepatic disease, sleep duration, residence, and annual income demonstrated significant associations with depressive symptoms.

**Table 2:** Associations between variables and depressive symptoms.

Exposure	Depressive symptoms OR (95%CI) *p*-value
Hobby	
No	1.0
Yes	0.70 (0.65, 0.76) <0.0001
Age	1.02 (1.01, 1.02) <0.0001
AGE1.GROUP	
Low	1.0
Middle	1.24 (1.14, 1.34) <0.0001
High	1.52 (1.40, 1.64) <0.0001
Sex	
Female	1.0
Male	0.49 (0.46, 0.53) <0.0001
Marital status	
No spouse	1.0
Married with spouse	0.56 (0.51, 0.61) <0.0001
Education	
Illiterate	1.0
Primary	0.65 (0.60, 0.71) <0.0001
Middle	0.45 (0.42, 0.49) <0.0001
College or higher	0.30 (0.27, 0.34) <0.0001
Self-reported health	
Good	1.0
Fair	2.70 (2.46, 2.96) <0.0001
Poor	8.07 (7.27, 8.96) <0.0001
Hypertension	
No	1.0
Yes	1.33 (1.24, 1.42) <0.0001
Diabetes mellitus	
No	1.0
Yes	1.40 (1.28, 1.52) <0.0001
Heart disease	
No	1.0
Yes	1.81 (1.67, 1.95) <0.0001
Stroke	
No	1.0
Yes	2.18 (1.92, 2.48) <0.0001
Arthritis	
No	1.0
Yes	2.40 (2.25, 2.57) <0.0001
Dyslipidemia	
No	1.0
Yes	1.36 (1.26, 1.46) <0.0001
Hepatic disease	
No	1.0
Yes	1.57 (1.39, 1.77) <0.0001
Sleep duration (h)	0.78 (0.77, 0.80) <0.0001
Sleep duration.GROUP	
Low	1.0
Middle	0.39 (0.36, 0.43) <0.0001
High	0.24 (0.22, 0.27) <0.0001
Residence	
Urban	1.0
Rural	1.80 (1.68, 1.93) <0.0001
Annual income (CNY)	1.00 (1.00, 1.00) <0.0001
Annual income.GROUP	
Low	1.0
Middle	0.67 (0.62, 0.72) <0.0001
High	0.34 (0.32, 0.38) <0.0001

Results in table: OR (95%CI) *p* value.

### The association between hobby engagement and depressive symptoms

Table 3 presents the associations between hobby engagement and depressive symptoms using different analytical approaches: crude Analysis, multivariable Analysis, and propensity-score analyses. In the crude analysis, hobby engagement was associated with a significantly lower odds of depressive symptoms, with an odds ratio (OR) of 0.70 (95% CI: 0.65, 0.76, *p* < 0.0001). In the multivariable analysis, after adjusting for all covariates, the OR was 0.89 (95% CI: 0.82, 0.97, *p* = 0.0109). When adjusting for the propensity score (PS), the OR was 0.91 (95% CI: 0.84, 0.99, *p* = 0.0204), and with PS (smooth), the OR remained 0.91 (95% CI: 0.84, 0.99, *p* = 0.0206).

**Table 3:** Associations between hobby engagement and depressive symptoms in the crude analysis, multivariable analysis, and propensity-score analyses.

Analysis	Depressive symptoms	*P*-value
No. of events/no. of patients at risk (%)		<0.001
Hobby engagement	1286/4073 (31.57%)	
No hobby engagement	4754/11984 (39.67%)	
Crude analysis—Odds ratio (95% CI)	0.70 (0.65, 0.76)	<0.0001
Multivariable analysis—Odds ratio (95% CI)		
1: Adjust for all covariates	0.90 (0.82, 0.98)	0.0115
2: Adjust for PS	0.91 (0.84, 0.99)	0.0204
3: Adjust PS(smooth)	0.91 (0.84, 0.99)	0.0206
Estimate of exposure effect using IPTW—Odds ratio (95% CI)		
ATT	0.91 (0.84, 0.98)	0.0151
ATC	0.92 (0.85, 1.00)	0.0523
ATE	0.92 (0.85, 0.99)	0.0362

IPTW, Inverse Probability of Treatment Weighting Using the Propensity Score. ATT, average treatment effect for treated. ATC, average treatment effect for control. ATE, average treatment effect for all. Note for models: 1: Adjusted for indicator of any missing, age (smooth), sex, marital status, education, self-reported health, hypertension, diabetes mellitus, heart disease, stroke, arthritis, dyslipidemia, hepatic disease, sleep duration (h; smooth), residence, annual income (CNY; smooth). 2: PS: propensity score calculated by indicator of any missing, age (smooth), sex, marital status, education, self-reported health, hypertension, diabetes mellitus, heart disease, stroke, arthritis, dyslipidemia, hepatic disease, sleep duration (h; smooth), residence, annual income (CNY; smooth).

### Stratified analysis for the association between hobby engagement and depressive symptoms

**Figure 2:**
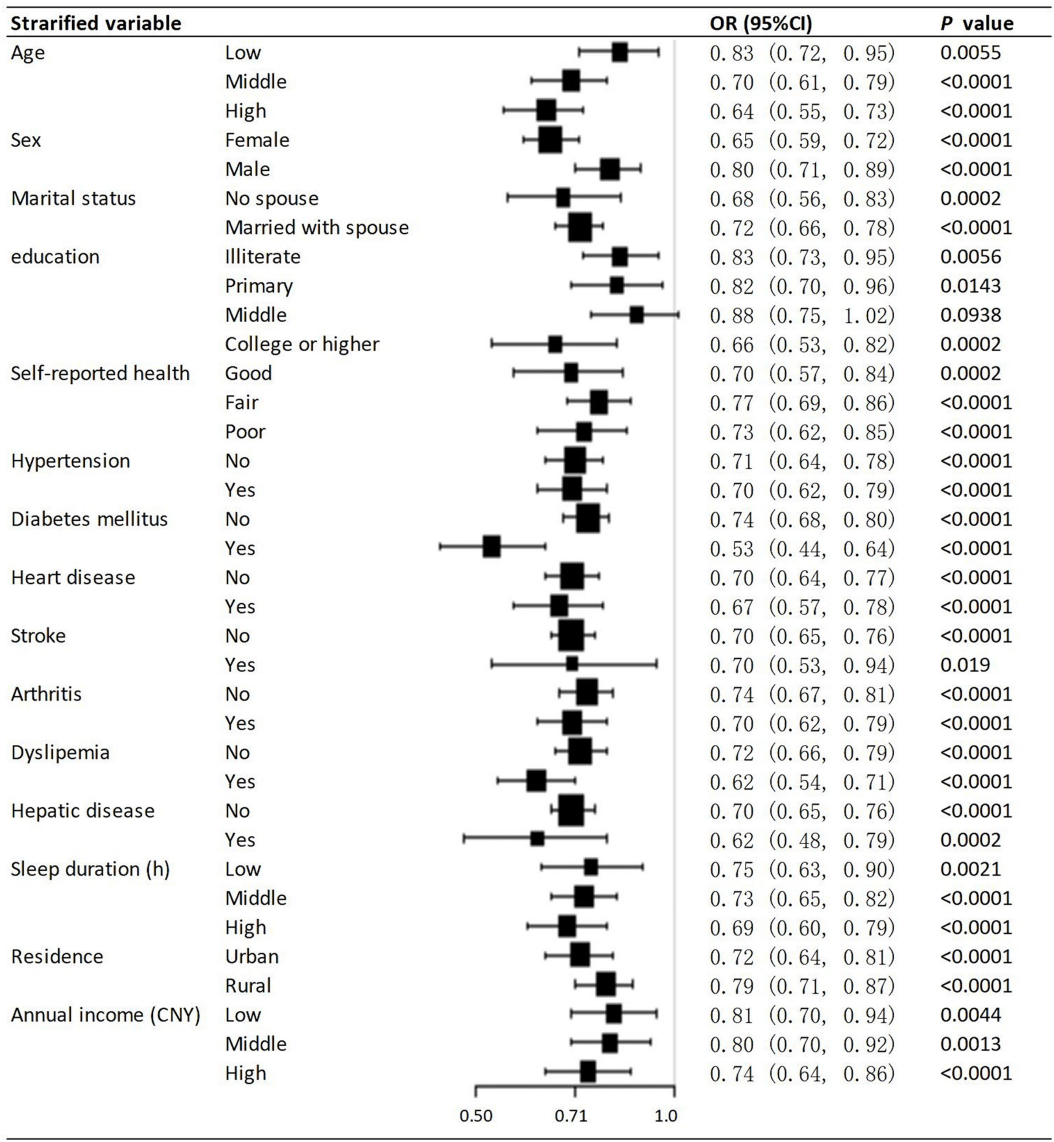
Stratified associations between hobby engagement and depressive symptoms CI, confidence interval; HR, hazard ratio.

Figure 2 presents the results of the stratified analysis examining the effect of hobby engagement on depressive symptoms across different covariates. The odds ratios (ORs) for depressive symptoms associated with hobby engagement were 0.83 (95% CI: 0.72, 0.95, *p* = 0.0055) for the low age group, 0.70 (95% CI: 0.61, 0.79, *p* < 0.0001) for the middle age group, and 0.64 (95% CI: 0.55, 0.73, *p* < 0.0001) for the high age group. The ORs for depressive symptoms were 0.65 (95% CI: 0.59, 0.72, *p* < 0.0001) for females and 0.80 (95% CI: 0.71, 0.89, *p* < 0.0001) for males. Other stratified covariates such as marital status, education, self-reported health, hypertension, diabetes mellitus, heart disease, stroke, arthritis, dyslipidemia, hepatic disease, sleep duration, residence, and annual income also showed significant associations between hobby engagement and depressive symptoms (Figure 2).

### Sensitive analysis

The sensitivity analysis yielded results consistent with those of the main analysis. Firstly, Table 3 demonstrates consistent associations between hobby engagement and depressive symptoms across various analyses. Using IPTW analyses, the average treatment effect for the treated (ATT) was 0.91 (95% CI: 0.84, 0.98, *p* = 0.0131), the average treatment effect for the control (ATC) was 0.92 (95% CI: 0.85, 1.00, *p* = 0.0574), and the average treatment effect for all (ATE) was 0.92 (95% CI: 0.85, 1.00, *p* = 0.0382). The results from crude, multivariable, and propensity-score analyses consistently show that hobby engagement is linked to lower odds of depressive symptoms. These results consistently indicate that is associated with a reduced risk of depressive symptoms across various analytical methods, highlighting the robustness of the observed association. Secondly, the direction of the effect size in each stratum is consistent in Figure 2, indicating that the association between hobby engagement and depressive symptoms is stable. Furthermore, we generated an E-value to assess the sensitivity to unmeasured confounding. The primary findings were robust, unless there was an unmeasured confounder with an OR greater than 1.31. Sensitivity analysis was performed in people with hypertension (*n* = 9,819), and the results were consistent with the core results, as shown in the Supplementary material.

## Discussion

This cross-sectional study found that hobby engagement was associated with a reduced risk of depressive symptoms in a large sample of middle-aged and older Chinese adults. The major finding was that after adjusting for covariates, the odds ratio was 0.89 (95% CI: 0.82–0.97). Propensity-score analyses also supported these findings, with the odds ratio being 0.91 (95% CI: 0.84–0.99, *p* = 0.0204). To the best of our knowledge, this is the first study to use the propensity score analyses to investigate the relationship between hobby engagement and DS in among Chinese middle-aged and older adults. However, further studies are required to confirm these findings.

Multiple studies have emphasized the significant impact of engaging in various leisure activities and artistic pursuits on alleviating depressive symptoms in the older adult. Older individuals often face limitations in participating in social activities due to declining physical abilities, leading to increased feelings of loneliness and isolation, which are closely linked to depression and cognitive issues (27). Our study shares certain similarities with previous research on the factors associated with depressive symptoms in the older adult, while also contributing new insights. Both studies focus on individuals aged 50 years and above. Our study investigates middle-aged and older Chinese adults, whereas the previous study targets older adult populations in South Africa. Both studies employ a cross-sectional design, enabling the examination of associations at a specific point in time. Additionally, both studies assess depressive symptoms as a primary outcome, although they utilize different definitions and measurements. Furthermore, both studies emphasize the significance of social engagement and activities in the prevention of depressive symptoms among older adults, underscoring the necessity for further research in their respective contexts. Nevertheless, there were notable discrepancies in the exposure variables. The objective of our study was to investigate the relationship between engagement in leisure activities and the occurrence of depressive symptoms. After adjusting for potential confounding variables, our findings indicated an odds ratio of 0.89. In contrast, previous research examining difficulties with social participation reported higher rates of depression in individuals who encountered these challenges (OR = 1.639) (27). Furthermore, our study employed propensity score analysis to substantiate its findings, reporting an odds ratio of 0.91. In contrast, previous studies have relied on multivariate analyses to demonstrate that depression is associated with an increased likelihood of difficulty in social participation. The disparate outcomes may be attributed to the fact that the studies were conducted in disparate cultural contexts, which may exert an influence on the factors associated with depression and the role of social engagement and hobbies in their respective societies. In conclusion, while both studies address key issues of depression in older adults and highlight the role of social engagement, they differ in terms of specific exposure factors, statistical methods, and contextual effects.

Research has shown that social participation plays a crucial role in combating depression among the older adult, especially during challenging times such as the COVID-19 pandemic, where psychosocial support and engagement in daily activities are essential for mental well-being (28). Moreover, studies have delved into the specific types of social participation and their effects on depression in the older adult, emphasizing the need to promote social engagement to reduce depressive symptoms effectively (29). Our study assessed hobby engagement through a questionnaire, capturing social activities in the past month. A binary indicator (yes or no) was created based on the responses. Activities included playing ma-jong, chess, or cards, attending a community club, participating in sports or social clubs, engaging in community-related organizations, doing voluntary or charity work, and attending educational or training courses. Our study found that hobby engagement was associated with a reduced risk of depressive symptoms in a large sample of middle-aged and older Chinese adults, which is consistent with previous research.

Furthermore, the prevalence of depressive symptoms among the older adult has been linked to factors such as social activities, with research indicating variations based on gender and urban versus rural settings, highlighting the need for tailored interventions to address specific demographic needs (22). The results demonstrated that the OR of the relationship between hobby engagement and depression in middle-aged and older men was 0.65 (95% CI: 0.59, 0.72). In middle-aged and older adult women, the OR value of the relationship between hobbies and depression was 0.88 (95% CI: 0.71, 0.89), indicating that the direction of effect was the same in both men and women. This suggests that hobby engagement was associated with a lower incidence of depression. The OR values for the urban and rural areas were 0.72 and 0.79, respectively. It should be noted that the present study does not address gender or residence differences as a research hypothesis. This topic could be the subject of further investigation.

Studies have also examined the impact of chronic illness, living arrangements, and ethnic backgrounds on depression among the older adult. For instance, research has shown that frail older adult individuals are at a higher risk of experiencing depression, emphasizing the importance of physical activity and exercise therapy in managing depressive symptoms and enhancing quality of life (30). In our study covariates were selected according to their clinical significance, including demographic characteristics, comorbidities, and general data for the following covariates: age, sex, marital status, education, self-reported health, hypertension, diabetes mellitus, heart disease, stroke, arthritis, dyslipidemia, hepatic disease, sleep duration, residence, and annual income. A variety of statistical methods are adopted to obtain the independent role of hobby engagement and depressive symptoms after taking into account the influence of the above covariates.

The rationale for selecting sleep duration as a variable in the model for this study was based on the critical factor of enhancing sleep quality as a means of combating depression. A substantial body of research has demonstrated a robust correlation between sleep quality and depressive symptoms, indicating that enhancing sleep can markedly alleviate depressive conditions. For example, it was demonstrated that poor sleep quality is associated with an increased risk of depressive symptoms, thereby underscoring the significance of sleep quality as a determinant in the onset of major depression (31). This relationship is further supported by, who noted that negative emotions negatively impact sleep quality, thereby initiating a vicious cycle that exacerbates depressive symptoms (32). The results of our study indicate that there is a statistically significant inverse relationship between sleep duration and depressive symptoms, with an odds ratio of 0.78 (95% CI: 0.77, 0.80). This finding is consistent with the results of previous research.

In the older adult, a lack of sufficient physical activity is regarded as a contributory factor in the development of several types of frailty. The findings of Zhang et al. indicate that regular physical exercise can not only prevent cognitive and functional decline but also reduce the incidence of various types of frailty (33). Concur, noting that the critical role of nutrition and physical activity in the prevention of chronic disease is well-established. Indeed, they highlight that improving these lifestyle behaviors can significantly reduce the burden of disease in older age groups (34). Considering the above reasons, we choose self-reported health, hypertension, diabetes mellitus, heart disease, stroke, arthritis, dyslipidemia, and hepatic disease as covariates in the model for this study.

The relationship between nutritional status and cognitive function is also worthy of note. Studies have demonstrated a significant correlation between malnutrition and cognitive decline, particularly in the older adult, and optimal nutritional status is regarded as a crucial element in the prevention of cognitive decline (35). Illustrate this with a systematic review by Cacador et al., which indicated that a decline in nutritional status was closely associated with a decline in cognitive and functional ability, emphasizing the significance of nutrition in geriatric care. The present study did not collect data directly related to nutritional status, although information on comorbidities and economic status was gathered as covariates. The lack of direct data on nutritional status represents a limitation of this study.

Theoretically, according to social integration theory, engaging in diverse leisure activities can enhance an individual’s social connections, which is beneficial for mental health (36). Empirical evidence corroborates this assertion. For instance, a study revealed that participation in multiple leisure activities and extended periods of exercise can mitigate the risk of depression in older adults (36). Furthermore, a longitudinal study of older adults in the UK demonstrated that participation in hobbies was associated with a reduction in depressive symptoms, and this relationship remained significant after controlling for other variables (37). A number of studies have indicated that participation in art and creative activities (such as music, painting, etc.) is associated with a reduction in depression, with the effect being more pronounced for certain types of activities (23) For instance, a study revealed that older adults who engaged in artistic pursuits exhibited a reduction in depressive symptoms over a two-year period (23).

### Study limitations

A common problem in observational studies is unmeasured confounders. As seen in Table 1, compared to the subjects in the hobby engagement group. Significant differences were observed among the hobby engagement groups in terms of age, sex, place of residence, marital status, level of education, annual income, self-reported health status, hypertension, diabetes mellitus, heart disease, stroke, arthritis, dyslipidemia, hepatic disease, duration of sleep, and depressive symptoms (all *p* < 0.05). These differences may be indicative of unmeasured confounders, such as income and medical insurance, which may affect the risk of depressive symptoms. Although we adjusted for possible confounding factors, including age, sex, marital status, education, self-reported health, hypertension, diabetes mellitus, heart disease, stroke, arthritis, dyslipidemia, hepatic disease, sleep duration, residence, and annual income. Additional limitations of our study include missing data for some variables. Nevertheless, we used contemporary methods to deal with missing data to minimize bias.

Secondly, another limitation of our study is that, as a cross-sectional study, it can only establish association rather than causation. The temporal relationship between hobby engagement and depressive symptoms cannot be definitively determined, which is a significant limitation given the implications of the study. Future longitudinal or experimental studies that would help clarify causal relationships and determine the effectiveness of different types of hobby engagements as interventions for reducing depressive symptoms in older adults. To illustrate, research interests may include, but are not limited to, the following areas: Chinese painting, humanistic painting, visual perception, folk art and folk art (38, 39).

Another limitation is related to the fact that the diagnosis of depressive symptoms was based on a self-reported questionnaire, which may introduce the possibility of misclassification. The use of the CES-D 10 scale to measure depressive symptoms was appropriate; however, reliance on self-reporting can introduce bias, particularly in a population where stigma may influence the willingness to report mental health issues. The results indicated a lower incidence of depression, yet there was no evidence to suggest that this bias influenced the relationship between hobby engagement and depressive symptoms.

Furthermore, the lack of information on interventions during the initial stabilization period could have influenced the risk of depressive symptoms. It is important to note that the potential effects of these interventions would likely bias toward null, leading to an underestimation of the association between hobby engagement and depressive symptoms risk.

Finally, it is acknowledged that the study participants were middle-aged and older Chinese adults who were referred to, which may limit the generalizability of the findings to other populations.

## Conclusion

The study analyzed data from the CHARLS database and identified 16,057 participants. It found hobby engagement was associated with a reduced risk of depressive symptoms in a large sample of middle-aged and older Chinese adults. These findings highlight the significance of addressing factors related to hobby engagement in the context of age-related health conditions.

## Data Availability

Publicly available datasets were analyzed in this study. This data can be found at: http://charls.pku.edu.cn/.

## Footnotes

^1^http://charls.pku.edu.cn/

^2^www.empowerstats.com

^3^http://www.r-project.org
