# Supplementary material for: The association between hobby engagement and depressive symptoms among Chinese middle-aged and older adults: evidence from the China health and retirement longitudinal study
Source: Front Public Health. 2024 Sep 27;12:1450358. doi: 10.3389/fpubh.2024.1450358 (PMC11466789; doi:10.3389/fpubh.2024.1450358)
Supplement: Supplementary file 1 [file Table_1.doc]

Supplementary material

# Supplemental material S1

**Table S1** Associations between hobby engagement and depressive symptoms in the Crude Analysis, Multivariable Analysis, and Propensity-Score Analyses in participants without hypertension (n = 9819) .

| Analysis | depressive symptoms | *P* value |
| --- | --- | --- |
| No. of events/no. of patients at risk (%) |  | <0.001 |
| hobby engagement | 750/2552 (29.4%) |  |
| No hobby engagement | 2688/7267 (37.0%) |  |
| Crude analysis — Odds ratio (95% CI) | 0.71 (0.64, 0.78) | <0.0001 |
| Multivariable analysis — Odds ratio (95% CI) |  |  |
| 1: Adjust for all covariates | 0.88 (0.79, 0.99) | 0.0272 |
| 2: Adjust for PS | 0.90 (0.81, 1.00) | 0.0452 |
| 3: Adjust PS(smooth) | 0.90 (0.81, 1.00) | 0.0401 |

Note for models:

1: Adjusted for indicator of any missing, age(smooth), sex, marital status, education, self-reported health, diabetes mellitus, heart disease, stroke, arthritis, dyslipemia, hepatic disease, sleep duration (h)(smooth), residence, annual income (CNY)(smooth).

2: PS: propensity score calculated by indicator of any missing, age(smooth), sex, marital status, education, self-reported health, diabetes mellitus, heart disease, troke, arthritis, dyslipemia, hepatic disease, sleep duration (h)(smooth), residence, annual income (CNY)(smooth).
